# Nanoparticle/Core-Shell Composite Structures with Superior Optical and Electrochemical Properties in a Dye-Sensitized Solar Cell

**DOI:** 10.3390/nano12183128

**Published:** 2022-09-09

**Authors:** Siti Nur Azella Zaine, Norani Muti Mohamed, Mehboob Khatani, Muhammad Umair Shahid

**Affiliations:** 1 Chemical Engineering Department, Universiti Teknologi PETRONAS, Seri Iskandar 32610, Malaysia; 2 Centre of Innovative Nanostructures and Nanodevices (COINN), Universiti Teknologi PETRONAS, Seri Iskandar 32610, Malaysia; 3 Electrical and Electronic Department, Universiti Teknologi PETRONAS, Seri Iskandar 32610, Malaysia; 4 Department of Physics, Faculty of Science, University of Sialkot, 1-Km Main Daska Road, Sialkot 51040, Pakistan

**Keywords:** thin film, photoelectrode material, core-shell structure, scattering effect, dye solar cell

## Abstract

The dynamics of competition between kinetic electron generation and recombination have restricted the development of a higher-performance dye-sensitized solar cells (DSSC). The key to minimizing the competition is optimizing the nanostructures and thickness of the photoelectrode film. It has been reported that the optimum thickness of photoelectrode film to achieve high-performance efficiency is about 12–14 µm. In this study, a photoelectrode film, which is approximately 4 µm thinner compared with those previously reported and has improved performance efficiency, was successfully developed by using composite nanoparticles and core-shell structures. The fabricated DSSC shows an enhanced light scattering, improved dye absorption capability, and reduced electron recombination rate despite the thinner photoelectrode film. The synthesized elongated nanoparticle structure provides a larger surface area for anchoring more dye molecules. In addition, the micron-sized core-shell structures with different refractive indexes of the inner and outer material resulted in multiple refractions and closed-loop light confinement. The successful development of a high-performance thin photoelectrode film will lead to material and cost savings.

## 1. Introduction

The dye-sensitized solar cell (DSSC) is a photoelectrochemical cell with a similar mechanism to photosynthesis in nature. It has a very effective charge separation that allows for better performance in diffused light conditions. This has opened a new perspective on building integrated photovoltaics (BIPV) as glass walls and windows [1,2] and self-powered Internet of Things (IoT) devices, such as wireless sensor networks and wearable electronics [3,4].

**Figure 1:**
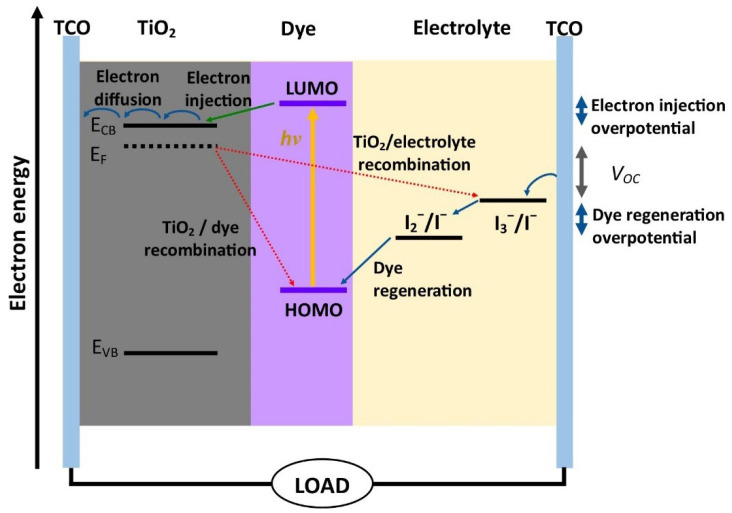
Schematic energy diagram and working mechanism of a DSSC.

A DSSC consists of three major components, namely, a transparent conductive substrate typically printed with TiO_2_ photoelectrode material and coated with a monolayer of dye sensitizer, redox couple electrolyte, and a catalytic conductive substrate (Figure 1). Light absorption occurs through the sensitizer embedded in TiO_2_, which acts like chlorophyll in leaves. When photon radiation strikes the dye molecules, the electrons become excited, are transferred to the conduction band of the photoelectrode and flow through to the external circuit. The original state of the dye molecules is restored through a regeneration process by the redox couple electrolyte [5,6,7].

Photoelectrode film is one of the essential elements in a DSSC. It serves as a photon capture, a medium of electron transport, and support for the dye sensitizer. However, a typical photoelectrode structure suffers slow electron transport and significant carrier loss through the recombination of the photogenerated electrons with the oxidized dye and the iodide species. Such dynamic competition between electron generation and recombination is the bottleneck restricting the development of higher efficiency DSSCs.

The dye sensitizer molecules play a vital role in absorbing light radiation. However, the molecules of dye sensitizer are too small to capture light radiation. In optimizing the light-capturing, the thickness of the photoelectrode film must be thicker than the light absorption length [8]. Realizing this necessity would ensure an adequate surface area for dye absorption and retain nearly 100% of the optical absorption characteristics. As a result, a micron-thick photoelectrode film is applied in the DSSC configuration to provide a large specific surface area for anchoring the dye and to confine the incident light radiance. However, considering the presence of charge recombination in an operational DSSC, the thickness of the photoelectrode films should not be larger than the electron diffusion length. Previous studies have reported that the optimum thickness of photoelectrode film is about 12–14 µm with an average particle size of about 10–25 nm [7,9,10,11]. Based on a study reported by Kang et al. [12], the performance of a DSSC is increased proportionally with the thickness of the photoelectrode films up to 15 µm. This was mainly due to the increase in surface area for anchoring the dye molecules. A recent study by Shah et al. also shows that the performance of solar cells increases as the thickness of the photoelectrode increases, due to the increase in the dye adsorption capability [13].

Introducing the light scattering effect in the photoelectrode film through the integration of large particles (>100 nm) is a promising approach to improving the capture of radiant photons [14,15,16,17]. The resonant scattering is most likely to occur when the size of the particles is comparable to the wavelength of the incident light [18]. Nevertheless, the introduction of larger particles has the drawback of reducing the internal surface area for dye adsorption, resulting in low light harvesting. On the other hand, utilizing smaller nanoparticles (~10 nm) is advantageous due to their ability to adsorb much more sensitizer. However, higher efficiency is hard to achieve due to the low light harvest in the red region [19,20]. A comprehensive study conducted by Chou et al. [21,22] on the effect of titania particle size on the performance efficiency of DSSCs shows the reduced size of particles results in reduced short-circuit current density. They attribute this to the variation of the facets and surface energy influenced by the different temperatures of hydrothermal treatments to produce the titania of varying particle sizes.

In this study, we aim to optimize the photoelectrode configuration by composing a double-layer configuration capable of absorbing many dye molecules, enhancing light scattering and reducing the recombination rate, thus enhancing the efficiency of a DSSC. The SiO_2_-TiO_2_ core-shell (STCS) structure [23,24,25,26,27,28] has been studied widely as a potential light scatterer due to the difference in the refractive index of core and shell that helps to provide better light scattering [23]. However, the STCS structures reduce the surface area, thus reducing the capability of the photoelectrode to anchor dye molecules [29]. Herein, we integrated the STCS structures with the nanoparticles TiO_2_ of right crystal facets to increase the capability of printed photoelectrode film to anchor dye molecules. Interestingly, a thinner photoelectrode film of 8 µm thickness with enhanced light scattering, dye absorption capability, and reduced recombination was produced successfully through the optimum configuration of composite nanoparticles and STCS structures.

## 2. Materials and Methods

### 2.1. Synthesis of TiO_2_ Nanoparticles

The TiO_2_ nanoparticle was synthesized through the sol-gel reflux method. All chemicals purchased from MERCK (Selangor, Malaysia) were used as received while deionized water was prepared using Milli-Q^®^ Direct (Merck Millipore, Selangor, Malaysia) water purification system ultrafiltered through 0.22 μm pore size filters. In this study, first an acidic water solution of pH 2 was prepared using deionized water and glacial acetic acid. An amount of zinc acetate dehydrates, and ammonium fluoride was then added to the solution. A 0.2 M titanium (IV) isopropoxide in absolute ethanol solution was added dropwise into the acidic water solution. The molar ratio of acidic water to the titanium isopropoxide was fixed at 110:1, and the concentration of salts was 1.0 mole over 100 moles of Ti^4+^ with the percentages of Zn^2+^ and F^−^ being 25% and 75%, respectively. The solution was then refluxed at 90 °C for 4 h. The precipitate was collected by evaporating the solution using a rotary evaporator at 80 °C with a speed of 50 rpm. The sample was then dried in a convection oven at 90 °C overnight.

### 2.2. Synthesis of SiO_2_-TiO_2_ Core-Shell Structures

The SiO_2_ core was synthesized through the Stober method with tetraethyl orthosilicate (TEOS) as a precursor, absolute ethanol as the solvent and 28–30% ammonium hydroxide solution as a catalyst. All chemicals were used as received without further purification. The de-ionized water was used to initiate the reaction. In the synthesis process, TEOS was added to 400 mL of ethanol containing 20% water solution. The solution was stirred for 30 min before adding an ammonium solution. The solution was then left for 18 h under slow stirring to ensure a complete hydrolysis process. The precipitate was then collected through centrifugation at a speed of 10,000 rpm for 15 min and washed with ethanol and deionized water.

The STCS structure was synthesized based on a previously reported study [29]. The synthesized SiO_2_ was dispersed in 50 mL of absolute ethanol. Then, 0.45 g of hydroxypropyl cellulose (HPC), 25 mL of absolute ethanol and 1.01 mL of deionized water were added to the dispersed SiO_2_ solution. The solution was stirred for at least 30 min to ensure that the HPC was completely dissolved in the ethanol solution. Then, the solution containing tetra-isopropyl orthotitanate in absolute ethanol was added dropwise into the solution containing the dispersed SiO_2_ prepared earlier. The mixture was then refluxed at 85 °C for 2 h under vigorous stirring. The precipitate was isolated using a centrifuge at 10,000 rpm for 90 min and then washed with ethanol and water. Finally, the collected sample was dried at 80 °C and then calcined at 500 °C for 2 h with a heating rate of 1 °C/min.

### 2.3. Fabrication of DSSC

Two types of photoelectrode paste were prepared in this study. The first paste consists of 100% synthesized nanoparticles while the second paste consists of a composite of the nanoparticles and 15% SiO_2_-TiO_2_ core-shell structures. First, 6 g of photoelectrode samples were dispersed with 20 mL of ethanol using an ultrasonic bath. Then, 1 mL of acetic acids and 5 mL of deionized water were added. The colloidal was stirred and dispersed again for another 1 h. In a different flask, a solution containing 10 wt% of ethyl cellulose solution was prepared by mixing ethyl cellulose and absolute ethanol. Then, 20 g of terpineol was added to the solution. After that, the colloidal was added to the solution. The slurries obtained were dispersed ultrasonically and then milled using an EXACT three-roll-mill at 150 rpm with 15 µm and 5 µm first and second roller gaps, respectively.

**Figure 2:**
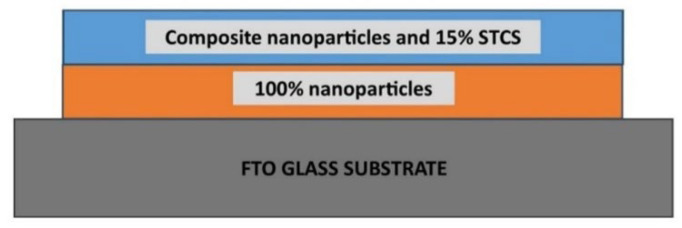
Illustration of the optimized configuration sample (synthesized sample). The bottom layer consists of the synthesized nanoparticles and the upper layer consists of the nanoparticles and 15% STCS composite.

A double-layer photoelectrode film was prepared by depositing the first layer with the nanoparticles paste and the second layer with the composite nanoparticle/STCS paste. The optimized configuration sample (synthesized sample) is illustrated in Figure 2. The printed films were then soaked overnight in the N719 dye solution and assembled in a sandwich layer with a catalytic conductive substrate and iodide/triiodide as electrolyte. Two commercial-based samples were prepared using commercial TiO_2_ nanoparticles purchased from Sigma Aldrich (commercial TiO_2_) and Greatcell Solar titania paste, 18NR-T for the bottom layer and 18NR-AO for the top layer (commercial paste). Details of the fabrication method have been reported previously [30,31].

### 2.4. Samples Characterization and Performance Verification

A high-resolution transmission electron microscope (HRTEM, TECNAI F20 X-Twin, PA, USA) was used to evaluate the particle size and morphology of the synthesized TiO_2_. An assessment of the particle shape and morphology of the synthesized SiO_2_ core, as well as the SiO_2_-TiO_2_ core-shell structure, was performed by using a variable pressure field emission scanning electrons microscope (FESEM, Zeiss Supra 55VP, Carl Zeiss, Oberkochen, Germany) with accelerating voltages of 5 kV at 100 kX magnifications. The FESEM images were taken with virtually no specimen preparation of overlayer coating. Scanning transmission electron microscopy together with energy-dispersive X-ray (STEM-EDX, Tecnai F20, FEI, PA, USA) was used for line analysis, and electron energy loss spectroscopy (EELS, Tecnai F20, FEI, PA, USA) was used for mapping analysis. In the analysis, the samples were dispersed in ethanol solution and drop-cast on a carbon-film-coated copper grid. The surface area, pore size and pore volume of the synthesized samples were evaluated using a surface area and pore size analyzer (SAP, Tristar 3020, Micromeritics, GA, USA).

FESEM was used to evaluate the thickness of the printed photoelectrode films. Their phases were evaluated using X-ray diffraction analysis (XRD, Empyrean, PANalytical, Malvern, UK) operated at 45 kV using a Ni-filtered Cu Kα radiation (λ = 1.54 Å) with step scan size of 0.01° and an omega of 2 in the range of 2θ of 10° to 80°. A UV-Vis spectrophotometer (UV-Vis, Cary 100, Agilent, CA, USA) was used to study the amount of dye absorbed by the photoelectrode films. In the testing procedures, the printed photoelectrode films with a cell size of 1 cm^2^ were soaked in N719 dye solution overnight. The samples were then rinsed with ethanol to remove excess dye and soaked in 5 mL of dilute ammonium hydroxide solution (10 mL of 30% NH_4_OH solution with 200 mL of deionized water). The desorbed dye solution was placed in glass cuvettes and tested for UV-Vis absorption spectra in the range of 800 nm to 200 nm with a dilute ammonium hydroxide solution as a baseline.

A universal photovoltaic test system, which is an integration of a solar simulator, Keithley 2420 source meter (Tektronix, OR, USA) and *I-V* testing software (*I-V* Software, Version 9, 2015, Michael Kelzenberg, CA, USA), was used to analyze the photovoltaic properties of the fabricated DSSC. The samples were analyzed at 100 mW/cm^2^ of simulated light of AM-1.5 radiation angle under variable load. The photocurrent action spectra analysis was performed using a 75 W Xenon lamp, and 100 mW irradiation in the range of 300–800 nm to verify the *I-V* analysis. The electrochemical properties of the cells were tested using Gamry Instruments PCI4-300 (Gamry Instruments, PA, USA) operating in two-electrode mode. The analysis was carried out in dark at various forwards bias voltages in the 300 kHz to 0.1 Hz of the frequency range and an AC modulation signal of 10 mV. Echem Analyst software (Version 7.8.2, Gamry Instruments, PA, USA) was used to fit the frequency-dependent impedance.

## 3. Results and Discussion

### 3.1. Physical Properties of the Synthesized Samples

**Figure 3:**
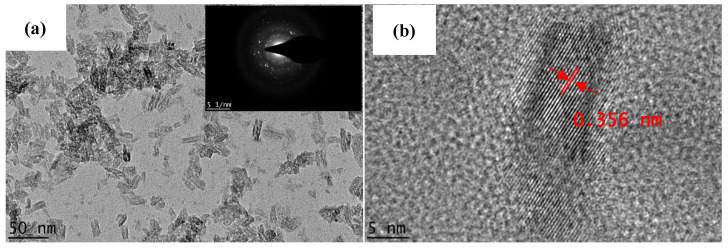
(**a**) TEM images of the synthesized TiO_2_ nanoparticles at a magnification of 88 kX and (**b**) high-resolution TEM image of the synthesized TiO_2_ nanoparticles at magnification of 620 kX.

Figure 3 shows the HRTEM images of the synthesized nanoparticles. HRTEM analysis shows that the sample is in the form of polycrystalline elongated nanoparticles. The higher magnification of Figure 3b indicates that the size of the synthesized nanoparticle was approximately 5 nm in width and 10 nm in length. The nanoparticles exhibit the lattice plane of the anatase phase with an interlayer spacing of approximately 0.356 nm [30,32,33].

**Figure 4:**
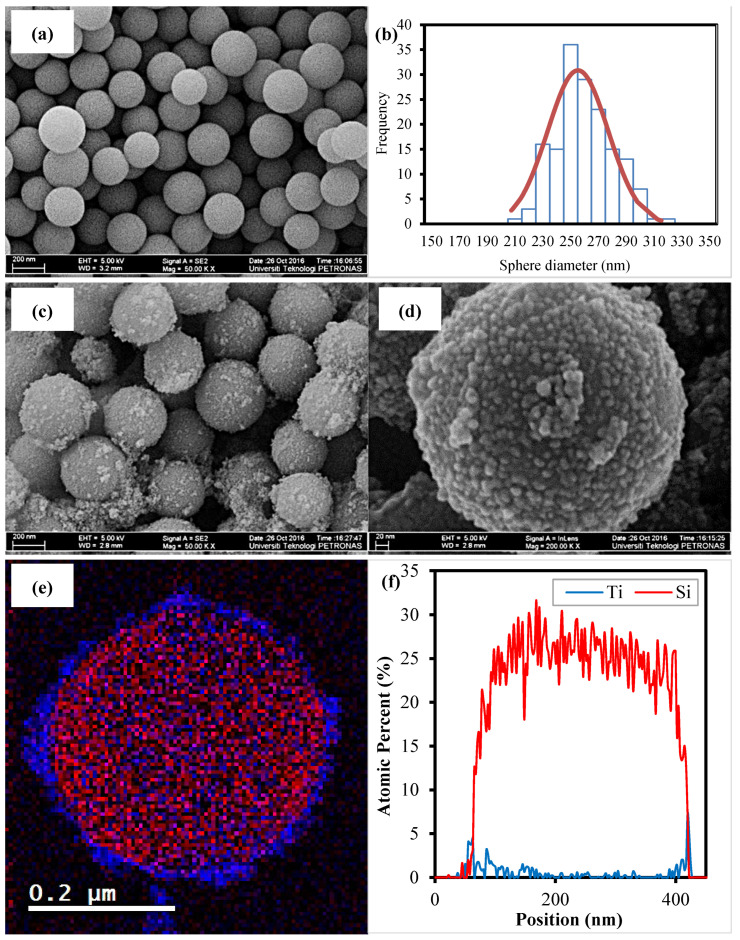
(**a**) FESEM image of the synthesized SiO_2_ sphere, (**b**) size distribution of the synthesized SiO_2_ sphere, (**c**) FESEM image of SiO_2_ coated with TiO_2_ at a magnification of 50 kX, (**d**) FESEM image of SiO_2_ coated with TiO_2_ at a magnification of 200 kX, (**e**) EELS dot mapping of the SiO_2_-TiO_2_ core-shell sphere and (**f**) EDX line scan of SiO_2_-TiO_2_ core-shell sphere.

Figure 4a,b show the FESEM images and the distribution curve of the synthesized SiO_2_ core prepared through the Stober method using 20% water in an absolute ethanol solution, 0.3 M of TEOS and 0.55 M of NH_4_OH. The image clearly shows that the synthesized SiO_2_ core is in the form of a highly dispersed well-rounded sphere with a mean size of about 255.0 nm and a standard deviation of 20.69 nm. Figure 4c,d show the FESEM images of the SiO_2_ coated with TiO_2_ nanoshell taken at two different magnifications of 50 kX and 200 kX. The FESEM images confirm that TiO_2_ were successfully coated on the surface of the SiO_2_ sphere. The high magnification FESEM image (Figure 4d) reveals that the TiO_2_ are in the form of nanoparticles with a size below 10 nm covering the surface of the SiO_2_, resulting in the rough surface morphology of the sphere. The electron energy loss spectroscopy (EELS) mapping analysis was utilized to reveal the distinct division between the SiO_2_ core and the TiO_2_ shell. Figure 4e is an image of the EELS analysis of a single SiO_2_-TiO_2_ core-shell sphere structure. In the image, the diameter of SiO_2_ is about 320 nm (denoted in red), while the shell thickness of TiO_2_ is about 10–20 nm (denoted in blue). The result was supported by the scanning HRTEM/energy-dispersion X-ray (STEM-EDX) line scan analysis, as shown in Figure 4f. In the graph of the atomic distribution of the EDX line scan, the broad red line represents the SiO_2_ core, and the Ti line width (blue line) represents the thickness of the TiO_2_ shell.

**Figure 5:**
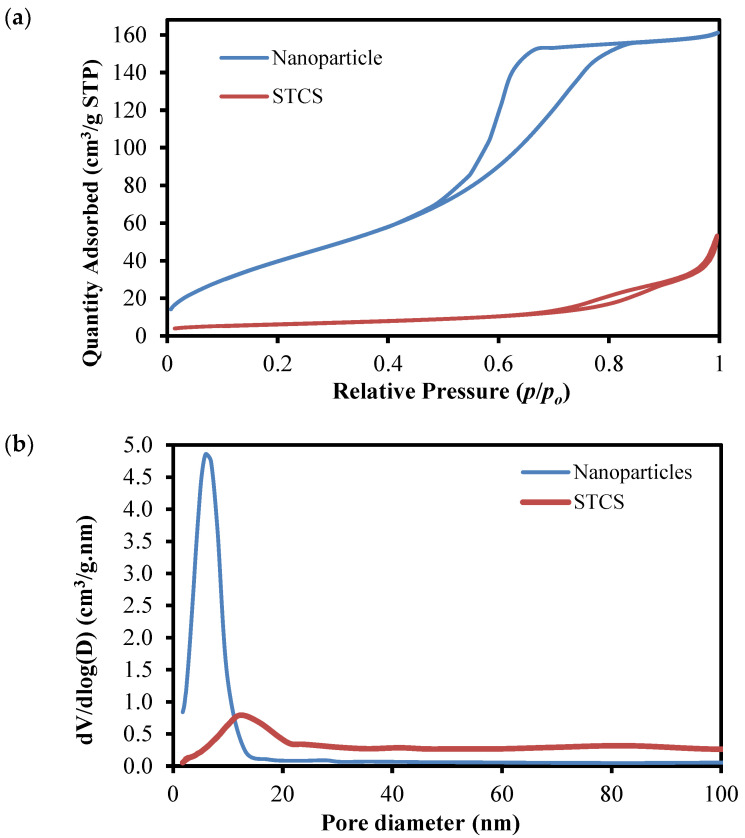
(**a**) Absorption-desorption isotherm of the synthesized TiO_2_ nanoparticles and STCS structures and (**b**) pore-size distribution of the synthesized TiO_2_ nanoparticles and STCS structures.

Surface area and pore size analyses were performed to evaluate the physical properties of the synthesized samples. Figure 5 shows the adsorption-desorption isotherm and pore size distribution (inset of Figure 5) of the synthesized TiO_2_ nanoparticles and STCS structure. The analysis shows that the synthesized nanoparticles sample exhibited a type IV adsorption isotherm with a type H2 hysteresis loop, indicating the presence of mesopore structures with well-distributed pores of 2–15 nm (blue line curve in inset of Figure 5). In contrast, the STCS sample exhibits a complex isotherm with a combination of type VI adsorption with a type H4 hysteresis loop, which does not exhibit any limiting adsorption at high *P*/*P_o_*. The properties represent stepwise multilayer adsorption with narrow slit-like pores. The type H4 usually signifies particles with irregular shapes and broad size distribution of voids. The pore size distribution curve of the STCS sample also shows the existence of a bimodal pore distribution of mesopores (2–50 nm) and macropores (>50 nm). These conditions could be explained by the microstructure of STCS consisting of a micron-size SiO_2_ sphere with a thin layer of shell composed of TiO_2_ nanoparticle structures. The presence of a mesopores distribution can be attributed to the nanoparticles TiO_2_ which made up the shell, whilst the macropores were attributed to the inter-sphere pores of the STCS structure. BET analysis shows that the nanoparticle sample exhibits a surface area of 158.35 m^2^/g, and the STCS sample exhibits a surface area of 21.82 m^2^/g.

**Figure 6:**
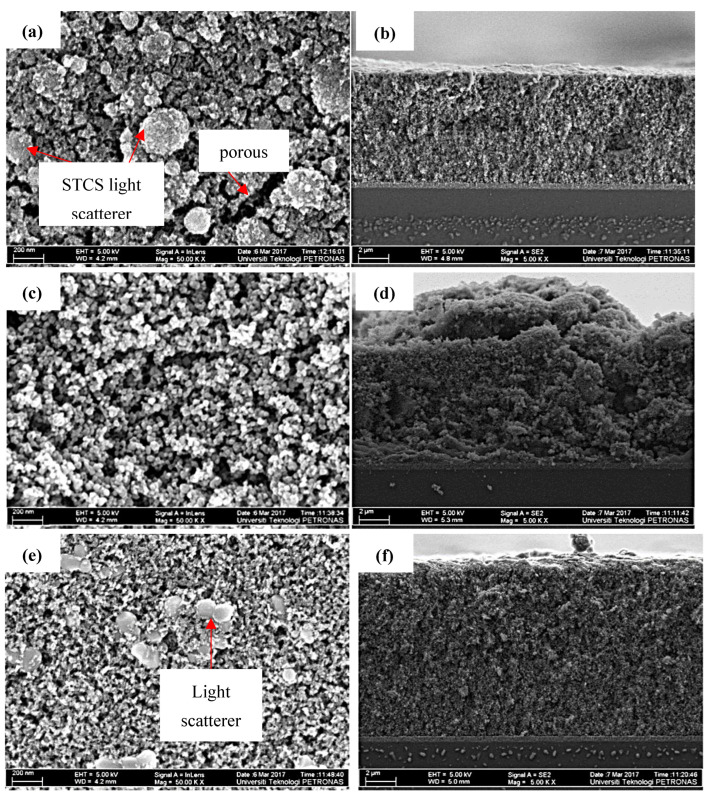
(**a**,**b**) FESEM images of top and cross-section view of the synthesized sample; (**c**,**d**) FESEM images of top and cross-section view of the commercial TiO_2_; and (**e**,**f**) FESEM images of top and cross-section view of the commercial paste.

The morphology of the printed photoelectrode films was analyzed using FESEM. Figure 6 shows the FESEM images of the top surface of the prepared photoelectrode film taken at the same magnification of 50 kX. Even though all samples were deposited with layers, the thickness of the synthesized sample was less (about 8 µm). On the other hand, the commercial TiO_2_ and the commercial paste produce a photoelectrode with a thickness of approximately 12 µm, as evidenced from the cross-section images from the FESEM analysis, as seen in Figure 6b,d,f. The FESEM images also show that the synthesized sample and the commercial TiO_2_ exhibit a porous structure, while the commercial paste is a densely packed photoelectrode film. Both the synthesized sample and the commercial paste were integrated with light scatterer particles in the top layer photoelectrode film.

**Figure 7:**
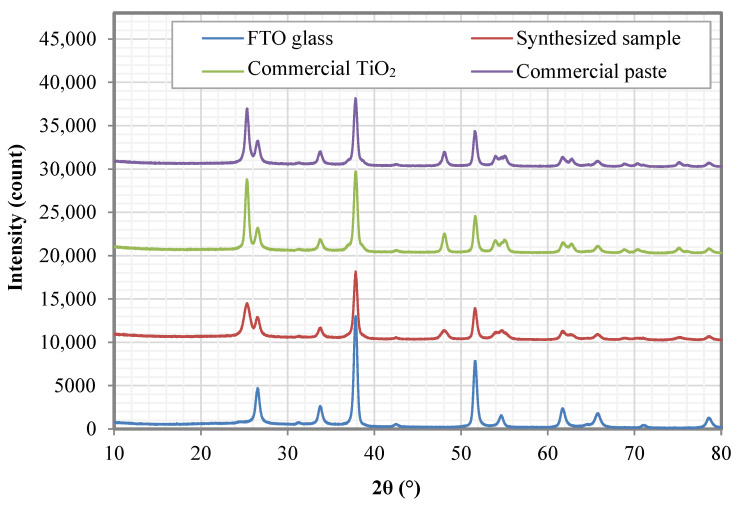
XRD analysis of the FTO substrate, synthesized sample, commercial TiO_2_ and the commercial paste.

The photoelectrode films were characterized using XRD analysis to determine their phase composition. Figure 7 shows the typical XRD spectra of the prepared photoelectrode film. The FTO substrate exhibit peaks at about 26.5°, 33. 7°, 37.8°, 51.5°, 61.6° and 65.6° corresponding to the tin oxide phase (PDF 00-046-1088), peaks at 31.2°, 51.5°, 61.6°, 65.6°, 70.9° and 78.5° corresponding to tin fluoride (PDF 01-085-2126) as well as the silicon dioxide phase (PDF 01-089-8937) at about 26.5°, 42.4° and 54.6°. The presence of these phases well matched the properties of the FTO glass substrate. In contrast, all the tested photoelectrode films exhibit extra peaks at 25.3°, 37.8°, 48.0°, 53.9°, 55.0°, 61.7°, 62.7°, 68.8°, 70.3° and 75.1° that represent the anatase phase with Miller indices of (101), (004), (200), (105), (211), (213), (204), (116), (220) and (215), respectively. The intensity of the FTO elements of the photoelectrode-coated substrate was due to the presence of a thick layer of photoelectrode film.

Table 1 shows the measured and calculated values for the XRD analysis of the (101) anatase plane. Comparing the intensity of the anatase peaks, the commercial TiO_2_ exhibits the highest intensity, corresponding to highly crystalline material, followed by the commercial paste and the synthesized sample. The commercial TiO_2_ also shows the narrowest of full-width half maxima (FWHM) which reflects the largest calculated crystallite size, followed by the commercial paste and the synthesized sample.

**Figure 8:**
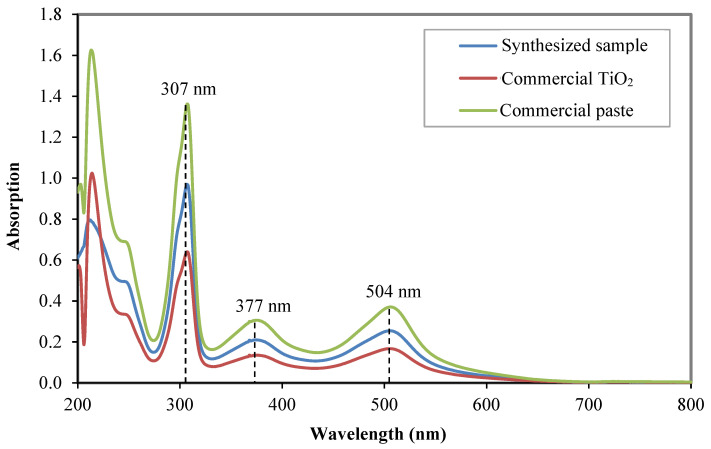
UV-Vis absorption spectra of desorbed dye solution of the synthesized sample, the commercial TiO_2_ and the commercial paste.

The capability of the photoelectrode films to absorb N719 dye molecules was evaluated based on the amount of absorbed dye. The number of desorbed dye molecules in an ammonium solution was tested using UV-Vis spectroscopy in the wavelength range of 200 nm to 800 nm. Figure 8 shows the absorption spectra of the desorbed dye solutions. The results show the typical absorption spectrum of N719 dye solution. The three peaks exhibited by all samples are at about 504 nm, 377 nm, and 307 nm, corresponding to the absorption spectra of the metal-to-ligand charge transfer (MTLC) transition in the visible region of N719 dye [5,34].

The analysis confirmed that the commercial paste exhibits the highest absorption capability followed by the synthesized sample and the commercial TiO_2_. The high absorption capacity of the commercial paste was due to the thick, densely packed, small-size nanoparticle film evidenced by the FESEM analysis. The presence of bigger particles as light scatterers in the commercial paste sample does not affect its dye absorption capability. The synthesized sample exhibits a considerably high dye absorption even though the samples were embedded with a low surface area of SiO_2_-TiO_2_ core-shell microstructure. The nanoparticle structures at the bottom layer of the film play a vital role in providing a large surface area for anchoring many dye molecules.

### 3.2. Performance Analysis of Fabricated DSSC

**Figure 9:**
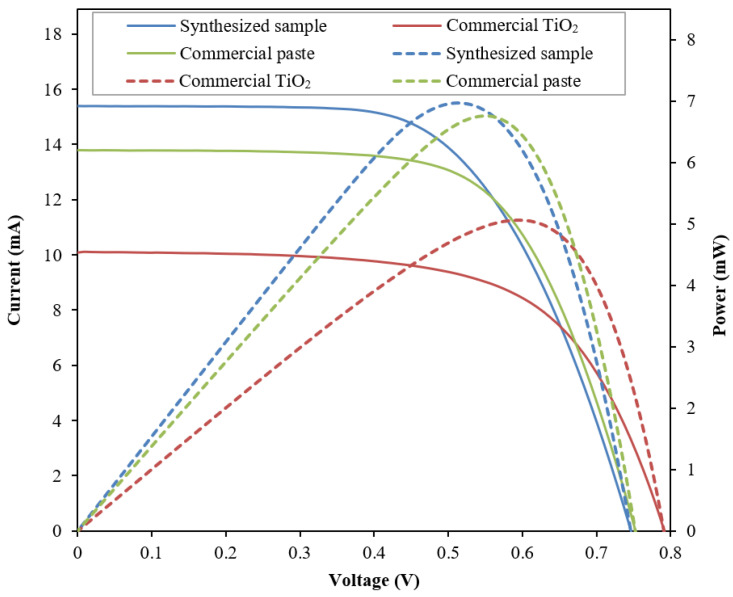
*I-V* and power plot of the fabricated DSSC integrated with the synthesized sample, the commercial TiO_2_, and the commercial paste as the photoelectrode film.

The performance of the fabricated DSSC integrated with the sample photoelectrode films was verified using a universal photovoltaic test system under 100 mW/cm^2^ intensity of Xenon lamp illumination at an AM-1.5 radiation angle, connected to a voltmeter and ampere meter (Model 2420, Keithly) with variable load. Figure 9 shows the *I-V* and power curve of the DSSC integrated with the synthesized sample, the commercial TiO_2_, and the commercial paste. Table 2 summarizes the photovoltaic properties of fabricated DSSC. The solid lines represent the *I-V* curve, while the dashed lines represent the power curve.

The analysis shows the synthesized sample exhibits the highest short-circuit current density, *J_sc_*, of 15.399 mA, followed by the commercial paste with 13.782 mA, and the commercial TiO_2_ with 10.080 mA. The high *J_sc_* of the synthesized sample contributes to the high-performance efficiency and maximum power produced by the fabricated DSSC. The synthesized sample exhibits the best performance efficiency of 6.88%. The improvement was calculated to be about 30% compared with the commercial TiO_2_ and 3.8% compared with the commercial paste. In terms of short-circuit current produced, the synthesized sample exhibits a more than 43.9% improvement compared with the commercial TiO_2_ and a more than 11.9% improvement compared with the commercial paste. However, the commercial TiO_2_ shows the highest open-circuit voltage, *V_oc_*, of about 0.793 ± 0.018 V, whereas the synthesized sample and the commercial paste have no significant difference (0.75 V). Nonetheless, the synthesized sample exhibits the lowest fill factor compared with the other samples. The low fill factor of the synthesized sample could be due to the small particle size, as evidenced in Figure 3, which leads to more defects, hence increasing the internal resistance of the fabricated cell.

**Figure 10:**
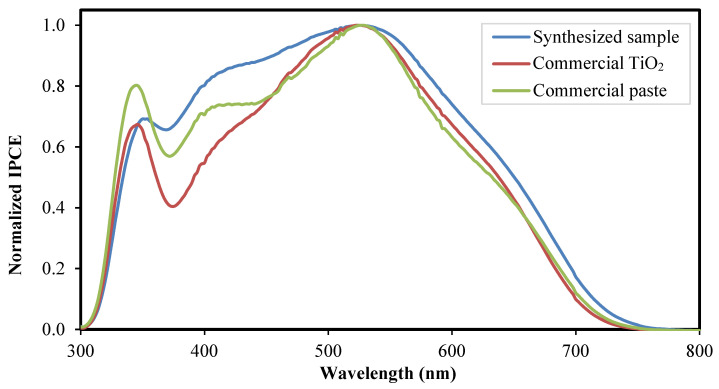
IPCE spectra of the fabricated DSSC integrated with the synthesized sample, the commercial TiO_2_, and the commercial paste as the photoelectrode film.

The current flows into the external circuit is measured by the difference between the amount of photo-generated current and the current loss in the diode and shunt resistance [35]. Three factors influence the value of *J_sc_*, namely, the light-harvesting efficiency (LHE), the electron injection efficiency of the photo-excited dye and the charge collection efficiency of the photoelectrode. The LHE depends on the molar extinction coefficient of the sensitized dye molecules, the number of the dye molecules anchored on the surface of photoelectrode TiO_2_ and the confinement of incident light. The *J_sc_* of the synthesized sample exhibits the highest value even though the sample has lower dye absorption capability compared with the commercial paste, as evident from the UV-Vis absorption analysis (Figure 8). This phenomenon can be explained by the enhanced light scattering of the core-shell structure. An incident photon-to-current conversion efficiency (IPCE) analysis was used to evaluate the effectiveness of the embedded SiO_2_-TiO_2_ core-shell structure as a scattering center. Figure 10 shows the IPCE spectrum of the tested DSSC.

The IPCE analysis was tested over the wavelength range of 300 nm to 800 nm. The spectra show peaks below 380 nm wavelength, corresponding to the energy band of the photoelectrode material of approximately 3.2 eV. The spectrum above 380 nm with a peak at about 530 nm corresponds to the N719 dye molecules. Based on the IPCE spectra, the synthesized sample exhibits the broadest peak compared with the commercial TiO_2_ and the commercial paste. The synthesized sample and the commercial paste exhibit a broad spectrum in the wavelength range of 380 nm to 530 nm. The broad spectrum corresponds to the presence of large particles, evident in the FESEM analysis. The particles of a size comparable to the incident wavelength act as light scatterers, extending the travel distance of an incident photon, confining the light radiance, and increasing the capability of the photoelectrode to capture light radiance. This phenomenon explains the increase in the short-circuits current of the optimized configuration cell [8,14,25].

**Figure 11:**
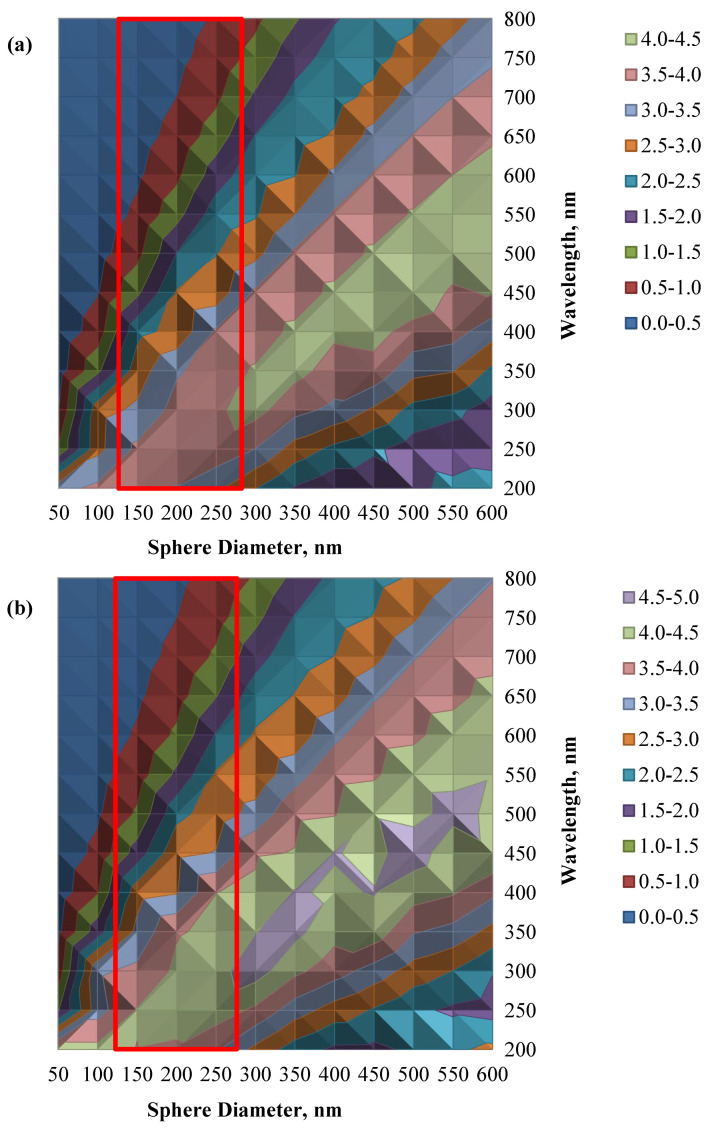
(**a**) The contour plot of the scattering efficiency, and (**b**) the contour plot of the extinction efficiency of the SiO_2_-TiO_2_ core-shell structure with a shell thickness of 15 nm.

To further understand the light scattering effect of the SiO_2_-TiO_2_ core-shell structures, a simulation study was conducted based on the Mie scattering theory of a single coated sphere [36,37]. In the simulation, three assumptions were made; the structure of the SiO_2_-TiO_2_ core-shell is circular, the light scattering by the dye molecules is insignificant due to their relatively small size (about 1 nm) compared with the wavelength of light, and the multiple scattering effects of the surrounding particles is neglected due to their considerably smaller percentage (15 wt%) and dispersed SiO_2_-TiO_2_ structure. Figure 11a,b show the contour plots of the simulated scattering and extinction efficiency of the STCS structure for the light wavelength of 200 nm to 800 nm for shell thicknesses of 15 nm.

The simulated results confirm that an optimum scattering efficiency occurs when the size of the light scatterers is comparable to the wavelength. Referring to Figure 11, it is clear that a light scatterer made up of SiO_2_-TiO_2_ core-shell sphere with a size in the range of 200 nm to 350 nm [23,25] with a shell thickness of about 15 nm [24] (marked in red line box) shows strong scattering and extinction efficiency in the wavelength range of about 300 nm to 550 nm. Light scatterers with a size less than 100 nm exhibit a weak light response if the incident wavelength is above 400 nm, whereas the large-size light scatterers (above 500 nm) demonstrate a weak scattering at a light wavelength below 350 nm. The simulation results also suggest that having a wide range of light scatterer sizes is essential to obtain optimum light scattering of all wavelength ranges in the visible region.

**Figure 12:**
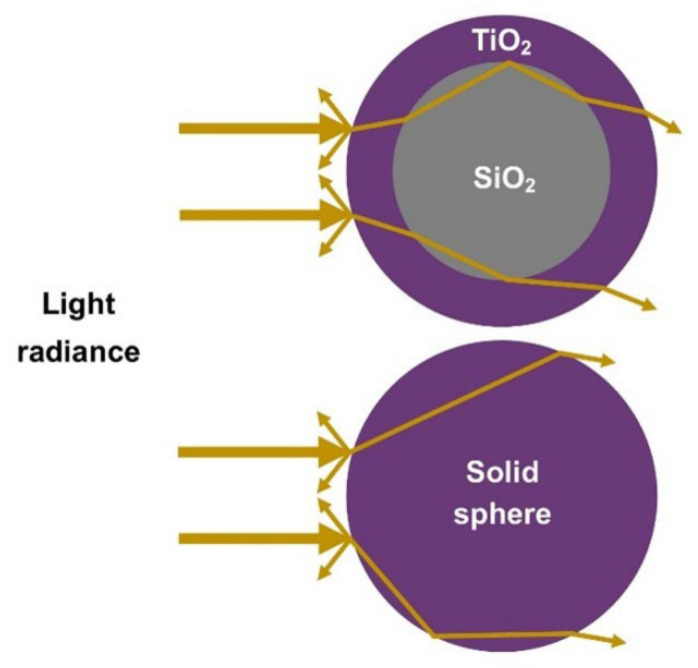
Schematic illustration of the incident light passing through a SiO_2_-TiO_2_ core-shell and a solid sphere structure.

The high scattering efficiency of the SiO_2_-TiO_2_ core-shell sphere structure is explained by the multiple light refractions of the incident light passing through the core-shell, as illustrated in Figure 12. The different refractive indexes of SiO_2_ and TiO_2_ materials caused the refraction of the incident light passing through the TiO_2_ shell, forming closed loops of light confinement, extending the distance of light travelled, and thus increasing the scattering efficiency. The simulated result is in good agreement with the IPCE analysis that explained the broad IPCE spectrum occurs in the wavelength of 380 nm to 530 nm.

**Figure 13:**
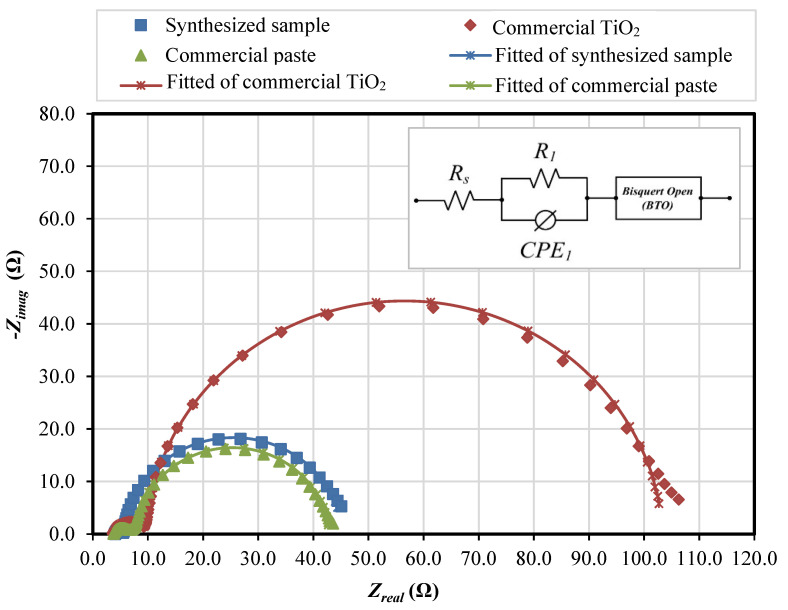
Nyquist plot of the impedance analysis of the synthesized sample, the commercial TiO_2_, and the commercial paste.

Electrochemical impedance spectroscopy (EIS) analysis was carried out to evaluate the electrochemical properties of the fabricated samples. Figure 13 shows the Nyquist plot of the fabricated DSSC fitted based on the Bisquert open model [38,39], and Table 3 shows the measured and calculated electrochemical properties such as transport resistance (*R_t_*), recombination resistance (*R_br_*), chemical capacitance (*C*_µ_), electron lifetime (*τ_n_*), reaction rate constant for recombination (*k*), electron diffusion coefficient (*D_n_*), effective diffusion length (*L_n_*) and steady-state electron density in the conduction band (*n_s_*).

The Nyquist plot shows that all fabricated cells exhibit two semicircles. The large semicircle represents the composite of electron transport in the photoelectrode materials and electron recombination between the photoelectrode–electrolyte interface. The small semicircle in the high-frequency region corresponds to the electron transport at the Pt counter electrode. Based on the analysis, the commercial TiO_2_ exhibits the largest recombination resistance, estimated from the large semicircle of the Nyquist plot. Both the synthesized sample and the commercial paste exhibit approximately the same recombination resistance. The low value of *R_br_* of both the synthesized sample and the commercial paste could be the reason for the low open-circuit voltage of the integrated DSSC. However, the transport resistance of the commercial paste is the highest. The low transport resistance of the synthesized sample allows the generated electrons to travel within the photoelectrode network before recombining with the oxidized species, thus reducing the rate of electron recombination. The EIS analysis shows that the recombination rate of the synthesized sample was approximately one-third that of the commercial paste and about half compared with that of the commercial TiO_2_. The low recombination rate of the optimized configuration cell leads to a longer electron lifetime.

As shown in Table 3, the calculated value of the steady-state electron density, *n_s_* of the synthesized sample is the highest, followed by the commercial paste and the commercial TiO_2_. High electron density leads to low transport and recombination and transport resistance [38]. The high electron recombination of the commercial paste increases its transport resistance, thus limiting the travel of electrons in the photoelectrode film towards the external circuit. This condition leads to a lower *J_sc_* value of the integrated DSSC even though the sample can anchor many dye molecules.

On the other hand, the commercial TiO_2_ shows the highest value of electrons diffusion, *D_n_*, among all the tested cells. One possible reason is the high crystallinity and large crystallite structure of the commercial TiO_2_ [38]. The high crystallinity corresponds to fewer defects, leading to an improvement of the transport properties of electrons and a reduction in electron recombination. This condition resulted in a better *V_oc_* for the commercial TiO_2_ compared with the other samples, as shown in Table 2. Interestingly, the *D_n_* of the optimized configuration cell is higher than the commercial paste even though the nanoparticles that made up the cell are much smaller. This outcome could be due to the thin photoelectrode film of the optimized configuration cell. The high electron diffusion in combination with the long electron lifetime of the optimized configuration cell leads to an improvement in electron diffusion length.

## 4. Conclusions

A thin photoelectrode-based DSSC of about 8 µm (4 µm thinner compared with a previously reported study) with enhanced light scattering, improved dye absorption capability and a reduced electron recombination rate was produced successfully. The photoelectrode film was developed by depositing a double-layer configuration with the bottom layer consisting of nanoparticles and the upper layer of composite nanoparticles and core-shell structures. The large surface area brought about by smaller nanoparticles has improved the capability of the photoelectrode film to anchor large amounts of dye molecules. The submicron size of the STCS structure of different refractive index of the inner core and the outer shell materials results in closed-loop light confinement and multiple refractions of the incident light, extending the travel distance of the incident photon within the photoelectrode film. The optimum configuration has successfully reduced the electron recombination rate by half, hence doubling the lifetime of electrons. The low recombination rate was due to the improved steady-state electron density, which leads to better trap state distribution, reduced electron transport resistance, thus promoting better electron diffusion, and improved electron lifetime. The successful development of a high-performance thin photoelectrode film will lead to material and cost savings.

## Figures and Tables

**Table 1:** The intensity, FWHM, d-spacing and estimated crystallite size of the synthesized sample, the commercial TiO_2_, and the commercial paste based photoelectrode films at the (101) plane of anatase phase.

Sample	Intensity (Count)	FWHM (*β*)	d-Spacing (*Å*)	Crystallite Size (nm)
Synthesized sample	2374.32	0.8253	3.5181	9.8
Commercial TiO_2_	4750.41	0.4529	3.5200	17.6
Commercial paste	4048.55	0.4767	3.5183	14.9

**Table 2:** Photovoltaic properties of fabricated DSSC integrated with the synthesized sample, the commercial TiO_2_, and the commercial paste as the photoelectrode film.

Sample	*J_sc_* (mA/cm^2^)	*V_oc_* (V)	FF	*η* (%)	*P_max_* (mW)
Synthesized sample	15.144 ± 0.33	0.747 ± 0.001	0.608 ± 0.003	6.880 ± 0.124	6.880 ± 0.124
Commercial TiO_2_	10.525 ± 0.386	0.793 ± 0.018	0.641 ± 0.013	5.289 ± 0.197	5.289 ± 0.197
Commercial paste	13.534 ± 0.353	0.753 ± 0.007	0.655 ± 0.004	6.626 ± 0.134	6.626 ± 0.134

**Table 3:** Electrochemical properties of the synthesized sample, the commercial TiO_2_, and the commercial paste.

Sample	*R_t_* (Ω)	*R_br_* (Ω)	*C*_µ_ (µ*F*)	*τ_n_* (*s*)	*k* (s^−1^)	*D_n_* (cm^2^ s^−1^)	*L_n_* (µm)	Con (Ωcm s^−1^)	*n_s_* (cm^−3^)
Synthesized sample	1.20	39.93	5.39 × 10^−3^	0.215	4.650	1.55 × 10^−6^	5.766	0.1485	10.80 × 10^17^
Commercial TiO_2_	3.66	93.80	1.10 × 10^−3^	0.103	9.718	2.49 × 10^−6^	5.062	1.00483	1.53 × 10^17^
Commercial paste	4.78	35.25	2.34 × 10^−3^	0.082	12.133	0.89 × 10^−6^	2.714	0.4919	3.26 × 10^17^

## Data Availability

Not applicable.
